# Equitable and specialised cardiovascular care in the Netherlands

**DOI:** 10.1007/s12471-024-01895-3

**Published:** 2024-08-15

**Authors:** Pim van der Harst

**Affiliations:** Department of Cardiology, Division of Heart and Lungs, University Medical Centre Utrecht, Utrecht, The Netherlands

In this issue, we feature two key studies and a review article to enhance our understanding of cardiovascular research and care in the Netherlands.

The first study, from the University Medical Centre Groningen (UMCG), examines women’s participation in clinical trials for atrial fibrillation. Khalilian Ekrami et al. found that women are well-represented, with a participation-to-prevalence ratio of 1.05, suggesting results are more generalisable [1]. Boersma’s commentary praises UMCG but also notes that the authors did not identify the success factors behind this participation rate [2]. Understanding these factors could help other studies achieve similar success.

The second study, from the University Medical Centre Utrecht (UMCU), highlights the success of left ventricular assist devices (LVADs) and a substantial increase in healthcare consumption among this patient population. Bosch reports that, over 6 years, LVAD implantations and related healthcare visits rose substantially [3]. This underscores the need for specialised, coordinated care to manage this growing patient population effectively. The study emphasises the importance of continuous improvement in clinical pathways and network care to reduce hospital stays and readmissions, using collaborations and e‑health solutions.

Advancements in telemedicine may provide solutions for managing heart failure (HF), as reviewed by Van Eijk et al. [4], who evaluated its effectiveness across various patient subpopulations. Their findings suggest telemedicine shows promise in reducing hospitalisations and improving outcomes, but its benefits vary among patient groups. The study calls for continuous real-world evidence to understand which HF patients benefit most, aligning with the LVAD study’s recommendation to optimise care through e‑health solutions.

These studies highlight the importance of equitable representation in research and the growing need for specialised care to meet the diverse needs of our patient populations.
